# MPLA Case 3: Don’t criticize me in public!

**DOI:** 10.1002/acm2.13334

**Published:** 2021-06-30

**Authors:** Kristi Hendrickson, Sara Kim, Cassandra Stambaugh, Mary Gronberg, Leonard Kim, Dongxu Wang, MPLA Cases Subcommittee

**Affiliations:** ^1^ School of Medicine University of Washington Seattle WA USA; ^2^ Department of Radiation Oncology Tufts Medical Center Boston MA USA; ^3^ Graduate School of Biomedical Sciences University of Texas MD Anderson Cancer Center Houston TX USA; ^4^ Department of Radiation Oncology MD Anderson Cancer Center at Cooper Camden NJ USA; ^5^ Department of Medical Physics Memorial Sloan Kettering Cancer Center New York NY USA

**Keywords:** case study, conflict management, leadership, MPLA, professionalism

## Abstract

This work of fiction re‐enacts a scenario in which a medical physics resident was not able to address a physics call during patient simulation and was criticized by the supervising faculty physicist in front of the team and the patient. The resident and the faculty agreed to meet afterwards to debrief the situation, in the hope of establishing a better working relationship. The intended use of this case, through group discussion, self‐study, or role‐play, is to encourage readers to discuss the situation at hand, inspire professionalism and leadership thinking, and allow the practice of conflict management. Facilitator's notes are available upon request to the MPLA Cases Subcommittee. This case study falls under the scope of and is supported by the Medical Physics Leadership Academy (MPLA), a committee in the American Association of Physicists in Medicine (AAPM).

## 1 INSTRUCTIONS FOR EDUCATIONAL USE

This MPLA case has a discussion format and a role‐play format, which can be used separately and individually.

The discussion format is a narrative case with accompanying discussion questions. A facilitator may distribute it to a group or audience for reading and discussion.

The role‐play format has differing backstory settings for an actor (the faculty or senior physicist) and a participant (the resident). The purpose of the role‐play format is for a student, a trainee, or a resident to practice skills in verbal communication and conflict management. Upon further expansion and adaptation by a user, this case can also be used to train a faculty or senior physicist on how to communicate with a trainee. The facilitator should only distribute one version of the backstory to the corresponding participant/actor and organize a conversational practice.

Name, gender, and other identities (if perceived) in this case are only representative. In either format, the facilitator has the discretion to change names and gender to be more representative of their specific context. In the role‐play format, it is recommended to choose the name and gender to match that of the participants.

The facilitator’s notes for both formats, as well as the editable version of the case, are available upon request to the MPLA cases subcommittee (https://www.aapm.org/org/structure/default.asp?committee_code=MPLACA).

## 2 DON’T CRITICIZE ME IN PUBLIC! (DISCUSSION FORMAT)

Andy had recently graduated and felt fortunate to match for residency. This residency had been his top pick since it was well‐known for its excellent clinical training, while his PhD program had provided very little clinical exposure. He was excited to continue his training at a residency program that would enable him to improve his clinical skills while still being able to continue his research. The first few months have been really hard on him. He clearly did not have as much clinical experience as his co‐resident and he felt like he had to work twice as hard to keep up with the residency expectations.

The residency recruited for two positions a year. As with most residency programs, the clinical skill level of the incoming residents could vary significantly. Therefore, the first few months of the program were spent orienting the new residents, assessing their clinical strengths and weaknesses, and providing basic clinical training. The expectation was that after the first few months of individualized training, each resident should be strong enough clinically that they could start having “Physicist of the Day” (POD) duty while paired with a faculty member.

Six months into the residency program, Andy was starting to feel more confident. He was just getting to the point where he was comfortable enough with how the clinic operated that he enjoyed POD duties. The only problem he was now facing was that certain faculty really weren’t great at teaching and didn’t seem to want him on their service for POD coverage. Since in most weeks there was at least one faculty member who liked to teach the residents with POD duty, Andy had started only picking those faculty to work with.

Due to the different clinical strengths of the residents and the faculty, the residents were allowed to choose which faculty member they would work with for POD coverage. Ideally, the residents would work with faculty who excelled in areas that they needed to further develop as well as with many different faculty so they could learn a variety of ways to approach clinical problems.

However, the program director, Dr. Kent, had noticed recently that the same faculty were picked frequently, while other faculty seemed to be avoided. This was starting to create tension within the department since teaching a resident, especially a new one, was more work, tended to make tasks take longer and could make clinical tasks requiring quick decisions even more stressful. Certain faculty had indicated that they thought that there was an unfair distribution of teaching responsibilities and that some faculty were purposefully not providing feedback or involving the residents in clinical decisions while POD so that the residents wouldn’t pick them.

One week, Dr. Kent decided to check in on the residents and their POD assignments based on the growing tension within the faculty. He walked into the resident room and found Andy sitting at his workstation.

“Hi Andy,” greeted Dr. Kent, “I wanted to check in on who you were planning to work with for POD coverage next week.”

Andy looked up from the paper he was reading. “Well, I initially was hoping to work with Dr. Hudson next week, but I just found out that he doesn’t have any POD duties since he is going on vacation. I need to go back to the faculty schedule and see who would be a good person to work with.”

Dr. Kent nodded. “OK. Why don’t we look at it together? I know you haven’t had POD duties for very long, but I’d like to make sure you are able to work with a variety of faculty members.”

Andy turned to his computer and pulled up the clinic coverage schedule. “It looks like Drs. Wu, May and Kenmore all have POD duty next week.” Andy continued, “Dr. May is really great at explaining machine issues so maybe I’ll work with her again.”

Dr. Kent peered over Andy’s shoulder, “Who of the three have you worked with the least?” he asked.

Andy paused briefly and responded, “I’ve only worked with Dr. Kenmore once so far. We got a POD call when he was trying to eat his lunch and he seemed quite frustrated that I interrupted him. I don’t think that he likes me working with him.”

Dr. Kent nodded again. To Andy he said, “It sounds like you may be avoiding Dr. Kenmore. I would hate for one poor interaction to diminish the learning opportunity you could have by working with Dr. Kenmore. He is a very good physicist. It would benefit you to work with Dr. Kenmore more. Do you think you could sign up for POD coverage with him the next week?”

Andy was hesitant but agreed. He should at least give it another chance, he thought. Maybe Dr. Kenmore was indeed a better teacher than he could have judged out of just one encounter.

The beginning of the next week went smoothly. He had the POD phone, he had checked in with Dr. Kenmore, and the morning was quiet. The POD calls they received were minor and while Dr. Kenmore wasn’t the most willing teacher, he clarified a few topics for Andy that had been confusing to him before.

Right around 3 PM in the afternoon, Andy got another POD call. It was from the CT simulator staff. They had a complicated simulation that they wanted the medical physics team’s input on. Andy immediately headed to Dr. Kenmore’s office to get him.

When Andy got to Dr. Kenmore’s office, the physicist’s door was open, but he wasn’t there. Andy waited in the office for about 5 min before the POD phone rang again. The patient was on the table of the CT simulator and they needed physics there immediately. Based on the urgency from the sim staff, Andy left a note on Dr. Kenmore’s desk and headed to the CT simulator.

As soon as Andy entered the simulation room, it was clear that the staff needed help. They were struggling to get the patient setup and the physician was clearly frustrated.

The physician, Dr. Cassidy, turned to Andy. “Well, can you fix this or not?”

Andy thought he could, as this situation appeared very similar to the one he had worked with Dr. Kenmore last time. This patient also clearly was having trouble keeping his shoulders down and was appearing anxious. As Andy walked over to take a better look at the situation, he asked, “does he need to be lying flat?” Dr. Cassidy barked, “I need to have his shoulders down and chin tipped so that I’m not treating through his shoulders or oral cavity to treat his neck. As long as that is achieved he doesn’t need to be totally flat, but none of the head cups we try are tall enough.” Andy thought that if he could get the patient lying flat, he could figure out the shoulder issue, but the patient wasn’t even close to where he needed to be. Dr. Cassidy started pacing and then asked “do you have any solutions?” As Andy racked his brain trying to remember what Dr. Kenmore had done previously, the faculty physicist walked into the simulation room.

The physician turned now to Dr. Kenmore. “I really need you to step in here. We can’t afford to have our workflow delayed like this.”

Dr. Kenmore stepped in and began working with simulation staff to appropriately setup the patient with a pliable head cushion, hand grips, and a long, open‐faced mask, as Andy observed. As they were finishing up the setup, Dr. Kenmore turned to Andy. “We have gone over this setup before. You should have been able to handle this, but you need to involve me prior to going off solo to POD calls so that if you can’t, you don’t delay clinical workflows.”

Andy saw the patient look at him as Dr. Kenmore spoke and immediately felt humiliation. When Dr. Kenmore turned back to the simulation staff, Andy left the room. Luckily, there were no more POD calls the rest of the day, and Andy managed to avoid Dr. Kenmore.

Later that night Andy received an email from Dr. Kenmore. “Andy, we need to talk about what happened today. You should have contacted me before going to the sim. It’s not OK for you to exert your own independence in this manner without guidance. I don’t like to see our reputation tarnished because a resident didn’t have good judgment.” Andy regretted agreeing to work with Dr. Kenmore, and reluctantly setup a meeting for the next morning to discuss the day’s events.

**Discussion** **Questions:**

1. As a trainee, what did Andy do well and what could he have done better in this situation?
2. As an educator, what did Dr. Kenmore do well and what could he have done better with this situation?
3. What contributing factors played a role into creating a negative interaction between Andy and Dr. Kenmore?
4. Examine the interactions of other players in this scenario. If you were the patient, what would be your takeaway? How could this have an impact (people, department, hospital)?
5. How might both Andy and Dr. Kenmore prepare for their meeting?
6. “Praise in public, criticize in private” is a quote from Vince Lombardi and discussed in the book *Radical Candor* by Kim Scott in reference to giving feedback. Do you think this is always appropriate? In this scenario, do you think it would have made a difference in the interaction between Andy and Dr. Kenmore?
7. What could be improved in this medical physics group communication practice to render such situations a thing of the past?

## 3 DON’T CRITICIZE ME IN PUBLIC! (ROLE‐PLAY FORMAT)

### 3.1 Participant scenario: Medical physics resident

I am a medical physics resident and 6 months into my 2‐year training. I came to the residency program after my PhD in medical physics with minimal clinical experience. Some residents have had more clinical experience before entering residency. The learning curve has been a bit steep, which has been a mild source of stress for me. By and large, I have been gaining confidence in the program, although I know I need a lot more clinical experience to gain a greater autonomy. I do take comfort in the feedback I have received so far that I have good judgment, strong theoretical medical physics knowledge, and a genuine interest in the clinical work.

I largely get along with the faculty and residents, although us newbies seem to be peripheral figures in the eye of some faculty. As a resident, I had to choose to work with a faculty of my preference for POD duty. I really wanted to work with Dr. Hudson this week, who has an excellent reputation as a teacher. But Dr. Hudson was not assigned as POD at all this week and, instead, I was paired up with Dr. Kenmore. I generally choose not to work with Dr. Kenmore because he is not a good teacher. My working relationship with Dr. Kenmore has been cordial albeit formal. Dr. Kenmore is someone who shows up and does the work but it is widely known that he makes little effort to get to know colleagues and trainees at a personal level.

One major responsibility I have to undertake now is to take calls on the POD phone, track down faculty, and consult on simulation setups. Typically, the faculty member is the one initiating a conversation on the situation. After a POD call this week, I walked by Dr. Kenmore’s office and he wasn’t there, although his office door was open. The last time when he wasn’t in his office, I tracked him down in the hallway as he was walking toward his office with lunch. When I told him about the POD call, he let out a big sigh, dropped off his lunch on his desk, and we walked to sim in complete silence. It was so uncomfortable as if I was punished for having interrupted his lunch. Well, that’s not my fault!

This time, I waited around Dr. Kenmore’s office. When the POD phone rang the second time, I proceeded to sim alone after leaving a note on his desk. I know the sim staff get very testy if I take too long to respond. The physicians and therapists were struggling with the setup. I proceeded to take a look at the sim. I knew we had a similar situation like this before but I couldn’t remember how Dr. Kenmore solved the issue. *“Well, can you fix it or not?”* I could hear the physician’s impatience in his voice. I started blushing as my mind drew blank. The patient looked at me, and I couldn’t tell whether it was out of empathy or anxiety. Soon Dr. Kenmore walked in. The physician said in a loud voice, “*We are not going anywhere here with this resident*. *We can’t* *afford to have our workflow delayed like this.”* Dr. Kenmore quietly worked on the sim and then turned to me and said,*”* *I showed you last time how to troubleshoot a set up like this. How many times do I have to repeat myself?”* I felt unjustly blamed for the situation and was totally humiliated in front of everyone. I walked out of the room.

Last night, Dr. Kenmore emailed me and asked to see me. I am in his office. I’m not sure how the conversation is going to go.

### 3.2 Actor’s backstory: Faculty

I have been a member of the medical physics faculty for 7 years at this institution. Previously I was an academic medical physicist with both research and clinical responsibilities at another institution. I largely enjoy my current roles with the exception of teaching responsibilities. I really didn’t receive formal preparation in how to teach and how to provide feedback to trainees to improve their performance. I must not be the only one feeling this way based on how the teaching loads seem to be shared in an unequal manner among faculty. In fact, it has been a bit of a sore point among faculty because of the perception that some people carry a larger share of teaching responsibilities. I do try to contribute to teaching as much as my schedule allows since that’s the whole point of being in a university setting. I am not sure if my Chief thinks I am a great team player, though.

I was told by my Chief that peers and leadership think I am unwilling to take on additional assigned projects needed by the department. Apparently, I have a reputation of going the great lengths to explain how busy I already am without bandwidth to take on more work. My peers have been frustrated with me because they ended up having to assume more work. My philosophy is if people have something to tell me, they should come to me directly without creating a secret trail of talking behind my back to the Chief.

I am the backup faculty POD today with Andy, the resident trainee. The resident responded to a call on the POD phone by going to the sim alone, which was not the right decision in this case. I saw a note left by the resident on my desk. The resident really should have waited for me before going solo. During sim, the resident was unable to answer questions about a special setup, frustrating the physician and therapists. The appointment with the patient was delayed, while the resident first tried to understand and solve the problem before calling me in to the situation. When I walked in, the lead physician barked at me, “*I really need you to step in here. We can’t* *afford to have our workflow delayed like this.”* I was irked that the physician lashed out at me in front of everyone.

I was able to give appropriate setup advice to the team and turned to the resident, who was standing by me, and said, *“I thought we went over a set up like this before. Also, you really need to involve me first before going solo*.” The patient probably was unaware that there was a problem but looked a bit puzzled by our interactions.

When I turned around, the resident was no longer in the room. I know the resident is in training and is supposed to learn from direct participation in the clinical environment. Trying to have residents gain gradual clinical autonomy has always been a delicate balance. I don’t expect residents to know all the answers when physics is called to the sim. But, I had shown this resident how to troubleshoot for a situation like the one we just saw, and I don’t understand why he couldn’t handle this situation with confidence. More importantly, he needs to understand that he should wait for the faculty physicist when responding to a POD call.

I emailed the resident and said, *“Andy, we need to talk about what happened yesterday. You should have contacted me before going to sim. It’s not ok for you to exert your own independence in this manner without guidance. I don’t like to see our reputation tarnished because a resident didn’t make a good judgement.”*

I am walking to my office where Andy is waiting for me.

## 4 AUTHOR CONTRIBUTION STATEMENT

KH and SK wrote the role‐play format and its facilitator guide. CS, MG, LK, and DW adapted the case into the discussion format and wrote the facilitator’s note. All authors have approved the final version.

